# Dyslexic savants

**DOI:** 10.4103/0972-2327.61288

**Published:** 2010

**Authors:** Sourabh Aggarwal

**Affiliations:** University College of Medical Sciences, New Delhi, India E-mail: drsourabh79@gmail.com

Sir,

I read the article “Taare Zameen Par and dyslexic savants”[1] by Ambar Chakravarty. It was quite an interesting article. It is good to observe that the entertainment industry is coming forward these days to raise their concern over social problems and provide remedial measures to them. It could not have come at a better time especially when the expectations of parents and competition pressure from peers is rising at an alarming level. The rate of suicide attempts and child abuse in post result time is quite worrisome. The mentally impaired children suffer the most. The extraordinary artistic talent of these dyslexic savants gets buried under the mounting pressure of success in the curricular field. These dyslexic savants have neuro-cognitive phenomenon of creativity in the midst of language disability. Though I agree that everyone cannot be as great as the Leonardo da Vinci or Sir Albert Einstein, but still parents need to learn a lot from this movie and this article. It is high time that such dyslexic savants are recognized at an early age and their art and interest properly channelized to fully nurture their extraordinary talent.
